# Influence of age on delayed surgical treatment of proximal femoral fractures

**DOI:** 10.1590/1413-785220152306149049

**Published:** 2015

**Authors:** Lisiane Pinto Gomes, Leandra Delfim do Nascimento, Tulio Vinicius de Oliveira Campos, Edson Barreto Paiva, Marco Antonio Percope de Andrade, Henrique Cerqueira Guimarães

**Affiliations:** 1 Universidade Federal de Minas Gerais, Multi-professional Residency in Elderly Health, Belo Horizonte, MG, Brazil.; 2 Universidade Federal de Minas Gerais, Faculdade de Medicina, Department of the Locomotor System, Belo Horizonte, MG, Brazil; 3 Universidade Federal de Minas Gerais, Hospital Risoleta Tolentino Neves, Belo Horizonte, MG, Brazil

**Keywords:** Hip fractures, Orthopedics, Aged, Comorbidity

## Abstract

**OBJECTIVE:**

: To investigate the influence of patients' age on the delay between diagnosis and surgical treatment of proximal femoral fractures

**METHODS:**

: This is a retrospective study, con-ducted at a tertiary university hospital, including all patients admitted with proximal femoral fractures between March 2013 and March 2014. The participants were categorized into four groups according to age levels. The groups were compared according to demographics, comorbidities, fracture type, trau-ma circumstances, and time between diagnosis and surgical procedure

**RESULTS:**

: One hundred and sixty one patients were included, 37 adults and 124 elderly. Among adults, the mean delay between diagnosis and surgical procedure was 6.4±5.3 days; among elderly the delay was 9.5±7.6 days. There was a progressive increase in the delay from the young-adults group through the elderly individuals (Kruskal-Wallis: 13.7; p=0.003)

**CONCLUSION:**

: In spite of being the patients most susceptible to complications due to surgical delay, the elderly individuals pre-sented the longest delays from admission to surgical treatment. **Level of Evidence III, Retrospective Study.**

## INTRODUCTION

Proximal femoral fracture (PFF) is a global level public he-alth issue.^1^ It occurs typically in individuals over the age of 60, especially in post-menopausal women,^2^ usually due to low-energy trauma such as falls to the ground.^3^ ^4^ However, this type of fracture can also be observed in young patients and, in these cases, it relates to high-energy trauma such as automobile injuries.^5^ ^6^

International recommendations suggest that patients with PFF undergo surgical treatment 24-48h after diagnosis.^7^ The be-nefit of early intervention seems to be more prominent in the elderly subpopulation considered fragile, to whom the resulting immobility of acute hip fracture can be devastating.^8^ However, it is not uncommon for the elderly individual to be admitted in adverse clinical condition, prone to postponement of surgery.^9^ Few studies have proposed to compare the characteristics of young and elderly patients with PFF.^10^ Moreover, we found no studies which have analyzed the influence of age in the time between hospital admission and surgery.

The *Hospital Risoleta Tolentino Neves* (HRTN) is a tertiary tea-ching hospital, associated to the *Universidade Federal de Minas Gerais* (UFMG) in Belo Horizonte, Brazil, which is responsible for emergency care of patients with traumatic and non-traumatic conditions in the populous northern axis of this metropolitan region. In this institution there are full structural conditions to offer the necessary surgery to correct any type of PFF. We report in this paper the results of a retrospective study that aimed to evaluate the association between age and various characteris-tics related to the occurrence and treatment of PFF.

## METHODS

A search for patients treated between March 2013 and March 2014, aged over 17 years, admitted under the International Classification of Diseases (ICD) record compatible with proxi-mal femoral fracture (S 72.0, S72.1, 72.2 S, S 72.8 and S 72.9) was performed in the electronic database made available from the Hospital's IT Department. Individuals with PFF admitted with codes different from the above mentioned were screened from the Surgical Center records. The study was approved by the Research Ethics Committee of UFMG on August 20^th^, 2014, under the protocol number 761,898.

It is worth noticing that in this Hospital the elderly admitted with PFF are daily tracked by an active search engine, and are served by a multidisciplinary orthogeriatrics team. These patients were submitted, within the first 24h of admission, to a pre-operative clinical assessment in order to provide pa-tients with surgical conditions as early as possible. Moreover, throughout the hospitalization period, these patients undergo a comprehensive geriatric assessment, dedicated to determine patients functional, neuropsychiatric, cognitive and motor as-pects, which allow the identification of factors and mechanisms associated with the occurrence of trauma in each individual.

Information concerning sociodemographic characteristics, cir-cumstances and characteristics of trauma, and clinical and neuropsychiatric comorbidities was obtained through a struc-tured collection instrument. The documented outcomes were: time elapsed between hospital admission and surgery, per-centage of cases solved within the institution, and in-hospital mortality rates.

### Statistical analysis

Patients were grouped according to age: Group I, adults aged 18-59 years, Group II, patients aged over 60 years. Group I was divided into Group A, formed by young adults aged 18-39 years, and Group B, represented by mature adults aged 40-59 years. The seniors group was subdivided into Group C, elderly aged 60-79 years, and Group D, elderly aged over 79 years old (very elderly).

Categorical variables were presented as proportions, and con-tinuous variables as mean ± standard deviation. According to *Kolmogorov-Smirnov* test a non-parametric distribution was observed for continuous variables. The groups were compared, therefore, through the chi-square tests for categorical variables and Kruskal-Wallis test for continuous ones. A value of *p* ≤ 0.05 was considered statistically significant.

## RESULTS

A total of 161 patients diagnosed with PFF were included in this study. Thirty-seven patients (22.98%) were aged between 18 and 59 years (adults). Of these, eleven (6.83%) belonged to group A, and twenty-six (16.41%) to group B. One hundred and twenty-four patients (77.01%) were older than 59 years (seniors) being forty-eight (29.81%) on group C and seventy-six (47.20%) on group D.

Table 1 shows the characteristics of the groups according to their distribution by gender, and types of fracture. There was a clear predominance of women in the elderly group (64.51% *vs*. 24.32%, p <0.0001). This phenomenon is even more pro-nounced in the group aged 79 years or older (very elderly) as compared to group C (72.36% *vs*. 52.08%; p=0.035). We identified no difference in gender distribution between adults' subgroups, in which there was a male predominance. In the adults' group there was a higher incidence of femoral neck fractures compared to the elderly group (51.35% *vs*. 31.45%, p=0.044), which showed a predominance of trochanteric frac-tures (62.09 *vs*. 35.13%, p=0.007).

Table 2 shows the causes of trauma. There was a higher fre-quency of traffic accidents in the adults' group as compared to the elderly group (29.72% *vs*. 2.41%, p<0.0001). From the age of 40 years on, fall to the ground becomes the most prevalent cause.

Table 3 shows the profile of comorbidities observed. The per-centage of victims who did not have any comorbidity was higher in the adult group (67.56% *vs.* 7.25%, p<0.0001), whereas multiple clinical comorbidities were observed more frequently in the elderly group (13.51% *vs.* 75%; p <0.0001). Parkinson-ism syndrome was found in 33.87% of the elderly. Additionally, dementia was reported in 49.19% of patients in this group, and was especially prevalent in group D as compared to group C (73.77% vs. 26.02%; p=0.009). It should be noticed, however, that adults received no formal geriatric assessment, i.e. were not submitted to motor and cognitive assessment.

As for the outcomes of the cases shown in Table 4, there were no differences regarding in-hospital mortality between groups. It is Important, however, to notice that a significant percentage of participants were transferred to other institutions for surgery, according to the healthcare network functioning of the city of Belo Horizonte. This outcome was more frequently observed in the elderly group (34.67% *vs.* 10.81%; p=0.009), especially in group D as compared to group C (42.10% *vs*. 22.91% p=0.046).

**Table 1:**
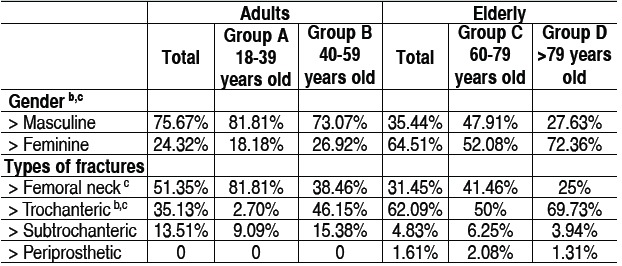
Distribution by gender and type of fracture.

a: A ≠ B, p < 0,05; b: C ≠ D, p < 0,05; c: Adults ≠ Elderly, p < 0,05

**Table 2:**
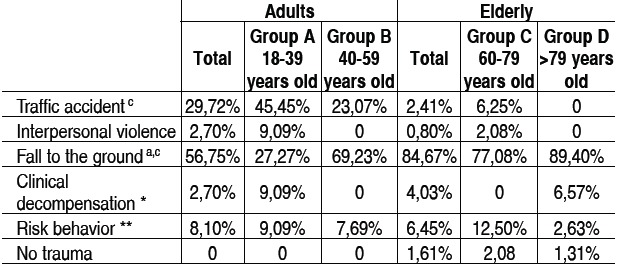
Characteristics of groups regarding causes of trauma.

*Hypoglycemia, epilepsy, etc.; ** Performing activities in height or house duties incompatible to cog-nitive and functional capacity; a: A ≠ B, p < 0,05; b: C ≠ D, p < 0,05; c: Adults ≠ Elderly, p < 0,05

**Table 3:**
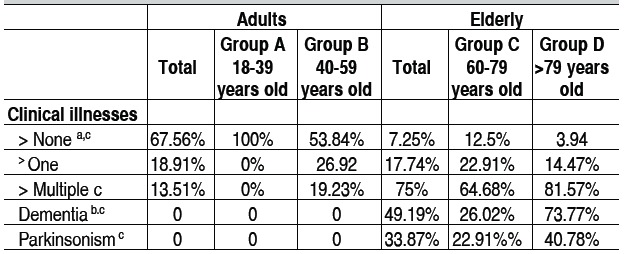
Comorbidity profile.

a: A ≠ B, p < 0.05; b: C ≠ D, p < 0.05; c: Adults ≠ Elderly, p < 0.05

**Table 4:**
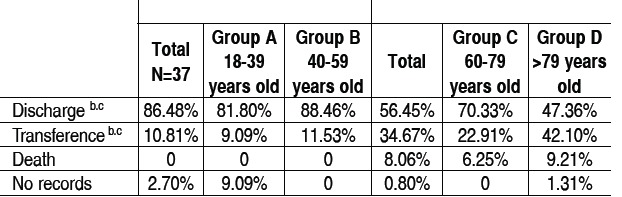
Hospital outcomes.

a: A ≠ B, p < 0.05; b: C ≠ D, p < 0.05; c: Adults ≠ Elderly, p < 0.05

**Figure 1:**
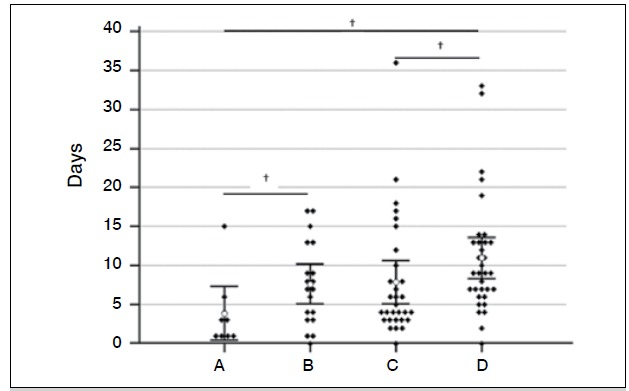
Time lapse between hospital admission by PFF and its surgi-cal correction according to the age groups studied (Kruskal-Wallis = 13.7, p=0.003; †p <0.05).

Figure 1 shows the distribution of time intervals (days) between hospital admission and surgical treatment of PFF among the four age groups. There was a clear progression in this interval according to age increase: group A, 3.88±4.51 days; group B, 7.63±5.32 days; group C, 7.87±7.49 days; and group D, 10.97±7.44 days (Kruskal-Wallis = 13.7, p=0.003). A *post-hoc* analysis between groups showed statistically significant differ-ences (p <0.05) between the elderly (group D), and participants in the groups C and A. The comparison between group D and B has not reached the threshold of statistical significance.

The disparate difference between elderly and adults regarding the delay observed between admission and surgery, we ana-lyzed the causes related to non-compliance of the proposed surgical planning among the different age groups (Table 5). The number of failures to perform surgery was significantly more frequent among the elderly compared to adults (59.99% *vs*. 27.03%; p=0.0007), especially in the group of older indi-viduals (71.42% *vs*. 41.18%; p=0.001). It is worth pointing out that some participants experienced changes on surgical plans more than once. Although not undergoing surgery for lack of medical condition or death was more frequently observed in the group of very elderly (10.71% *vs*. 0%; p=0.007), this situ-ation justified not performing surgery in only 11.11% of cases. The leading cause of non-compliance with surgical proposal was represented by logistical shortcomings in planning and realization of the surgery, as calendar outages within a reason-able time, unavailability of operating room on the scheduled day, insufficient anesthetists, lack of bed in intensive care unit (ICU) for the postoperative care, or lack of appropriate equip-ment for the surgery. This situation was observed in 53.33% of cases in the elderly group compared with 27.03% in the adult group (p=0.003), and was especially frequent in the very elderly (60.71% *vs*. 41.18%; p=0.04). It is noteworthy that the unavail-ability of ICU beds represented only 5.55% of the causes of non-surgical realization among the elderly.

**Table 5:**
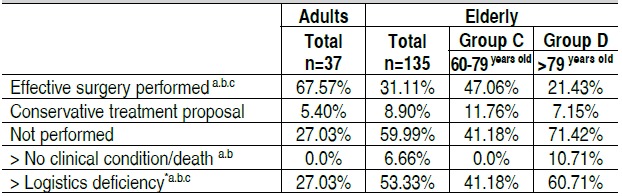
Surgical planning.

a: A ≠ B≠C, p < 0.05; b: C ≠ D, p < 0.05; c: Adults ≠ Elderly, p < 0.05. *Unavailability of agenda, Operating room, anesthetist, ICU bed and/or material/equipment

## DISCUSSION

The results showed that the PFF was more prevalent among the elderly, including a remarkable difference in gender distribution according to age, with a higher participation of men in the adult group and the opposite in the elderly. There were more traffic accidents and femoral neck fracture in the group of young adults, while in the other groups the most frequent cause of trauma was represented by falling to the ground, and the most observed type of fracture was trochanteric. Moreover, through extensive geriatric assessment, it was possible to identify a significant number of elderly people with neurodegenerative conditions, such as parkinsonism and dementia. The main fin-ding of the study, however, concerns the low surgical treatment efficiency in cases of elderly participants, characterized by a high transfer rate to other institutions and an unacceptable time delay between admission and surgery, considering the international recommendations for treating PFF.

Dissociation regarding gender distribution had been already described in a comparative study of trauma in the elderly and non-elderly at a teaching hospital of Curitiba, Brazil.^5^ The predominance of car accidents as a cause of trauma in very young people corroborates the impression on the association between masculine gender and greater exposure to risky acti-vities. The predominance of women in the elderly group, and especially in the very elderly, is a highly replicated finding in the literature^6^ and evokes various aspects related to female aging, such as longer life span, higher prevalence of osteo-porosis, dementia and disabilities.^11^

There was an increase in the frequency of falls to the ground with advancing age. Moreover, this mechanism was the main cause of fracture in all groups except in the group of young adults, a phenomenon reported in national and international studies on the topic.^12^ ^13^ It is noteworthy the frequency of falls to the ground in adults aged over 40. This result is in agreement with a study which assessed the demographic characteristics of victims of falls to the ground, where it was observed that 67.86% of patients were younger than 60 years,^14^ and reinforce the recommendation of a comprehensive geriatric assessment for those affected by PFF also in this age group.

The multidisciplinary evaluation strategy under geriatric medical orientation allowed the identification of dementia in 49.19% of elderly individuals, and parkinsonism in 33.87% of patients in this group. It is noteworthy that these figures were even more significant in the very elderly subgroup, in which dementia was observed in 73.77% and parkinsonism in 40.78% of participants. This binomial, cognitive impairment and motor disability, can be devastating to maintain a safe posture stability. Dementia causes impairment in several cognitive functions, and often compromises patients' critical judgment, motivating him/her to engage in activities for which he/she no longer meets the ability to execute safely.^15^ The incidence of parkinsonism increases remarkably with aging.^16^ Postural control, played by midbrain structures, is frequently affected in these individuals, and may be severely compromised, 17 especially when combined with the cognitive manifestations of dementia. Importantly, the identification of these conditions is an essential step for planning rehabilitative measu-res and those aimed at preventing future falls.

In the adults group, the average time between admission and surgery was 6.42 ± 5.3 days, and in the elderly group, 9.46 7.56 days. This result is in agreement with another national study, which included elderly aged 60-104 years, in which the mean time observed between admission and surgery was 9.35 

7.48 days.^18^ However, there is a clear non-compliance with international recommendations on the optimal time for surgical treatment of PFF, which postulate a maximum window of 48h between diagnosis and therapeutic intervention.^7^ As noted, this situation is not unique to the service reported here and can be observed in other Brazilian university hospitals with similar cha-racteristics as HRTN, suggesting that this is a commonplace phenomenon in our country. 

In the comparison between groups, it was observed that the resolution of cases was significantly higher in the group of adult patients at the expense of the elderly, and that the in-terval between admission and surgical treatment increased steadily over the four age groups. This finding is worrying since the elderly constitute the group most injured by the postponement of surgery.^8^ The main reason for patient transfer was related to the cancellation of surgery because of non--clinical reasons. The waiting time between admission and surgical scheduling was usually sufficient for most of the ad-verse clinical conditions to be circumvented. In fact, solving cases of young adults, markedly in higher number than the elderly's, points to weak institutional mechanisms to prioritize cases. The Association of Anesthetists of Great Britain and Ireland recommends the establishment of a specific trauma list for patients with hip fracture, priority based, including we-ekends and holidays, separately from the other surgical lists, besides non random allocation of anesthesiologist, given the complexity of these patients and the possibility of borderline clinical condition at the time of surgery.^19^ It should be noted that both recommendations are not followed by the institution where our data were collected.

Finally, it is important to point out the limitations of this study. Its retrospective nature limits the quality of the information ob-tained, especially regarding the details of the circumstances of trauma, which in many cases could not be rescued. The fact that only the elderly were subjected to formal geriatric asses-sment also limits the characterization of comorbidities in the group aged over 40 years, in which falls to the ground was the leading cause of trauma. It is possible that neurodegenerative conditions could also be found in these participants through specific diagnostic evaluation, contributing to the understanding of the causes of fall in this group. Finally, as a significant per-centage of the elderly participants were transferred to surgical treatment in other institutions, the analysis of mortality as an outcome is impaired in this study.

## CONCLUSION

We identified relevant differences between the age groups and various characteristics related to PFF. However, the therapeutic delay observed in the frailer group of patients was the most sa-lient and disturbing aspect of this work. Despite the fact that our institution relies on physical, personnel and equipment structure for the expeditious correction of PFF, these findings point to the need for institutional mechanisms for prioritizing this profile of pa-tients, according to established international recommendations. 
